# Observational Study of PD-L1, TGF-β, and Immune Cell Infiltrates in Hepatocellular Carcinoma

**DOI:** 10.3389/fmed.2019.00015

**Published:** 2019-02-08

**Authors:** Christian Ihling, Bartholomew Naughton, Yue Zhang, P. Alexander Rolfe, Eveline Frick-Krieger, Luigi M. Terracciano, Isabelle Dussault

**Affiliations:** ^1^Global Clinical Biomarkers & Companion Diagnostics, Merck KGaA, Darmstadt, Germany; ^2^Quantitative Pharmacology and Drug Disposition, EMD Serono, Inc., Billerica, MA, United States; ^3^Clinical Biomarkers & Companion Diagnostics Biopharma, EMD Serono, Inc., Billerica, MA, United States; ^4^Molecular Pathology Division, Institute of Pathology, University Hospital, Basel, Switzerland

**Keywords:** PD-L1, checkpoint inhibitor, CD8, immune cell infiltrates, hepatocellular carcinoma, HCC

## Abstract

**Introduction:** Hepatocellular carcinoma (HCC) typically develops in cirrhotic livers, with increased programed death ligand 1 (PD-L1) and transforming growth factor beta (TGF-β) activity implicated in immunosuppression.

**Methods:** In an observational study of HCC liver samples, we determined the incidence of PD-L1 and immune cell (IC) infiltrates, and signs of TGF-β activity. HCCs were characterized by the incidence and distribution of PD-L1^+^ cells, and CD8^+^, CD68^+^, and FoxP3^+^ infiltrating ICs in HCC and surrounding liver. Gene expression signatures (GESs) associated with TGF-β activity and ICs were evaluated by RNAseq.

**Results:** In non-neoplastic cirrhotic and non-cirrhotic liver, PD-L1 occurred on sinusoidal lining cells (mostly Kupffer cells), endothelial cells and ICs. In HCC, PD-L1^+^ tumor cells were rare. Most PD-L1^+^ cells were identified as ICs. CD8^+^, CD68^+^, and FoxP3^+^ ICs were associated with HCC, particularly in the invasive margin. CD8^+^ cell incidence correlated with PD-L1^+^ cells, consistent with PD-L1 being upregulated in response to pre-existing cytotoxic T-lymphocyte activity. TGFB1 mRNA levels and TGF-β activation GES correlated with the strength of the tumor-associated macrophage GES.

**Conclusion:** Inhibition of PD-L1^+^ ICs and TGF-β activity and their respective immunomodulatory pathways may contribute to antitumor effects in HCC.

## Introduction

Cancers develop through the acquisition of genetic and epigenetic changes that incidentally lead to the expression of novel antigens. These neoantigens are recognized in the course of immunosurveillance, enabling the elimination of many nascent tumors (1). To develop, tumors must therefore evade the immune system (2).

Compared with most other cancer types, hepatocellular carcinomas (HCCs) develop in an environment that is rather peculiar. The liver environment is intrinsically immunosuppressive, an adaptation that prevents the development of inflammatory liver damage secondary to routine exposure to foreign antigens derived from the gut (3). In addition, chronic inflammation associated with liver diseases (hepatitis B/C virus infection, alcoholic and non-alcoholic steatohepatitis) that typically precede HCC (4) leads to T cell exhaustion and exaggerated immunosuppression (5). HCCs therefore typically develop within a strongly immunologically suppressed liver environment. HCCs can additionally co-opt or enhance immunosuppressive mechanisms within the liver to survive and develop (1). The development of effective immuno-oncology therapies targeting HCC therefore requires specific understanding of the immunosuppressive characteristics of HCC in the contexts of the liver and liver diseases.

Important molecular components of the immunosuppressive network implicated in HCC include the checkpoint inhibitor programed death ligand 1 (PD-L1) and the immunosuppressive cytokine transforming growth factor beta (TGF-β) (1, 6–9). Together, these and other molecules modify the activities of multiple immune cells (ICs) including cytotoxic CD8^+^ T cells, regulatory CD4^+^ T cells (Tregs), natural killer (NK) cells, macrophages, dendritic cells (DCs), and myeloid-derived suppressor cell (3, 10).

PD-L1 is a ligand for PD-1 that is expressed on activated CD8^+^ T cells and some other ICs, such as macrophages. PD-L1 binding to PD-1 inhibits cytotoxic T cell activity and promotes other immuno-inhibitory effects (1). Yarchoan et al. report that PD-L1 expression is increased in HCCs relative to liver (11), while a recent study by Sia et al. has shown that 24% of HCCs have markers of an inflammatory response, with high PD-L1 levels (referred to as an “immune class”: tumors with significant enrichment of signatures identifying immune cells, i.e., T cells, cytotoxic cells, tertiary lymphoid structures, and macrophages; immune metagenes; and interferon gene signatures) (12). Furthermore, Calderero et al. have shown that PD-L1 expression by neoplastic cells and inflammatory cells in the tumor microenvironment (TME) of HCCs is significantly correlated with markers of tumor aggressiveness (13), and increased PD-L1 expression in HCC is associated with poor prognosis (6, 14) and disease recurrence (15).

TGF-β is a cytokine family with three members, TGF-β1, TGF-β2, and TGF-β3. TGF-β can exert both pro- and antitumor effects, depending upon cellular context, by modulating cell growth, migration, or phenotype (16, 17). TGF-β is often overexpressed in cancers including HCC (17), and HCC may promote TGF-β production by the TME (18). Acting through TGF-β receptors, TGF-β plays a pivotal role in the maintenance of self-tolerance, and can enable tumors to evade immune recognition. TGF-β suppresses CD8^+^ T cells, NK cells, and DCs, and promotes the development of Tregs (16, 18–20). TGF-β can also promote tumorigenesis by modifying the TME, supporting stromal modification, angiogenesis, and epithelial/mesenchymal transition (EMT) (21). Upregulation of TGF-β in cancers correlates with poor outcome (18). Within the immune class of HCCs with PD-L1 expression identified by Sia et al. and described above (12), a subclass expressed many of the genes regulated by TGF-β that are known to mediate immunosuppression.

To gain further insight into the potential immunosuppressive mechanisms present in HCC and the liver, we evaluated PD-L1, TGF-β, and infiltrating ICs in an observational study of a series of HCCs. The prevalence and distribution of PD-L1^+^ cells were assessed using semiquantitative immunohistochemistry (IHC), and double labeling was performed to characterize PD-L1-expressing cell types. Tumor-infiltrating ICs were evaluated by quantitative IHC, while TGF-β activity was assessed from gene expression signatures (GESs) determined from RNAseq data.

## Materials and Methods

### HCC Liver Samples

The study set consisted of 68 formalin-fixed, paraffin-embedded (FFPE) HCC resection specimens, from 68 patients, procured from Indivumed GmbH (Hamburg, Germany) and Agilent (Glostrup, Denmark and Santa Clara, California, USA). All patients who provided samples to Indivumed and Dako/Agilent gave written informed consent in accordance with the Declaration of Helsinki.

### Histology and IHC

To determine PD-L1 expression in HCC resection samples, serial 4–5 μm sections were stained with hematoxylin and eosin for histopathological assessment and were used for IHC. The diagnosis of HCC and the histologic subtype were confirmed independently by two board-certified pathologists (LMT and CI). Histologic grading and classification of HCCs were performed according to the World Health Organization (WHO) guidelines and the Edmondson & Steiner grading system (22). IHC was carried out using an Autostainer link 48 (Dako) with primary monoclonal antibodies recognizing PD-L1 (MKP-1A-73-10; PharmDx), CD8 (C8/144B; Dako), CD31 (JC70A; Dako), CD68 (PG-M1; Dako), FoxP3 (236A/E7; Abcam), and pan cytokeratin (AE1/AE3 Ventana). Envision FLEX HRP polymer and DAB^+^ (Dako) secondary reagents were used. For double labeling, the LabVision™ Multivision Polymer Detection System (ThermoScientific) was used according to the manufacturer's directions.

### PD-L1 Scoring

PD-L1 staining of non-neoplastic liver tissue was assessed qualitatively to evaluate PD-L1 expression in adjacent non-HCC parenchyma. PD-L1 staining of tumor cells was assessed qualitatively and semi-quantitatively by two board-certified pathologists (LMT and CI). Discrepant scores were discussed during joint examination and a consensus score agreed. The proportion of viable tumor cells with membrane staining of any intensity was determined by manual evaluation of the entire slide. The case was scored positive if ≥1% of the tumor cells were stained. The proportion of tumor area and contiguous peritumoral stroma occupied by IC with any degree of PD-L1 staining was also determined by semi-quantitative scoring.

### Quantitative Assessment of ICs in Different Compartments of HCC

To evaluate immune cell infiltration in HCC, the numbers of CD8^+^ (T cells), CD68^+^ (macrophages), and FoxP3^+^ (regulatory T cells) ICs were assessed quantitatively in the following tumor regions identified by two board-certified pathologists (CI and a pathologist employed by Mosaic Laboratories, Lake Forest, CA, USA): the whole tumor region (ALL), the area occupied by tumor; CT, the center of the tumor; and IM, the invasive margin (defined as the region extending 500 μm to either side of the border between the HCC and non-malignant cells). The frequency (% IHC^+^) of CD8^+^, CD68^+^, and FoxP3^+^ cells in each region was determined at a contract research organization (CRO; Mosaic Laboratories) using a computerized image analysis system. The incidence of ICs was defined as low (≤ median) or high (>median).

### RNAseq Analysis of FFPE Samples

GESs associated with TGF-β activity and ICs were evaluated by RNAseq. RNAseq was conducted at a CRO (Asuragen, Austin, TX, USA) on 50 samples, of which two failed quality control and were removed, leaving 48 samples in the final analysis. For each sample, the tumor was annotated by a board-certified pathologist (CI) and RNA was extracted from three whole-slide scrapes of 4–5 μm-thick sections with a tumor content >50%, using Recoverall Total Nucleic Acid Isolation Kit for FFPE (ThermoFisher Scientific). 200 ng of total RNA, quantified using RiboGreen® RNA reagent (Life Technologies), was depleted of ribosomal RNA using the Ribo-Zero Gold rRNA Removal Kit (Illumina). Strand-specific libraries were prepared using the NEBNext Ultra Directional RNA Library Prep Kit (NEB) and sequenced on an HiSeq2500 (Illumina) using 2 × 50 base-pair paired-end sequencing. Approximately 50 million reads per sample were obtained.

### Gene Expression Quantification

Sequencing reads were aligned against the hg19 reference genome using Bowtie2 version 2.2.3 (23). Gene expression was determined using RSEM version 1.2.31 with the Ensembl gene annotations (24). Two outlier samples with abnormally few detectable genes were excluded from further analysis. Transcript-per-million (TPM) values were upper-quartile normalized for further analysis.

### Gene Expression Signatures

GESs used in this study were: T-effector IFN-γ activity (25) HCC; subtype S1, as published by Hoshida et al. (26); EMT (27); tumor-associated macrophages (TAM) (28); CD8^+^ T cells (29); and TGF-β1-associated activity (Ingenuity Pathway Analysis, Qiagen). Gene signature compositions are provided in Supplementary Table 2. For a given GES, the strength of the signal in a sample was calculated as the sum of the log fold-increases relative to mean expression for all constituent signature genes in that sample. The strength of the GES in a sample was then normalized to the mean strength of the GES for the whole series.

CIBERSORT (29) was used to infer the frequencies of CD8^+^ T cells in a given sample and was used as a surrogate for CD8-associated activity.

### Statistical Analysis

Descriptive statistical analyses were performed using R version 3.3.1 (30). Correlation coefficients were calculated using the Pearson method. Significance was established using pairwise *T*-tests; *P*-values <0.05 were considered statistically significant. Plots were generated using the “R” package “ggplot2.”

## Results

### Samples

The characteristics of the 68 patients who provided samples are summarized in Supplementary Table 1.

### Histology, PD-L1 Expression, and ICs in HCC

Evaluation of tumor histology with hematoxylin and eosin staining revealed morphology typical of HCC in all 68 samples. They were categorized as low- to high-grade trabecular, pseudoglandular, or solid including mixed types with common cytoplasmic features (Supplementary Table 1).

In 16/68 cases extratumoral liver tissue was present, e.g., in eight cases we found non-cirrhotic tissue and in the remaining eight cases the extratumoral liver tissue showed cirrhosis.

In the eight cases of non-cirrhotic liver tissue adjacent to HCCs, liver architecture was largely normal. ICs were seen in all eight of these cases, principally in portal tracts, and to a lesser extent in the lobules, with variable frequencies.

**Figure 1:**
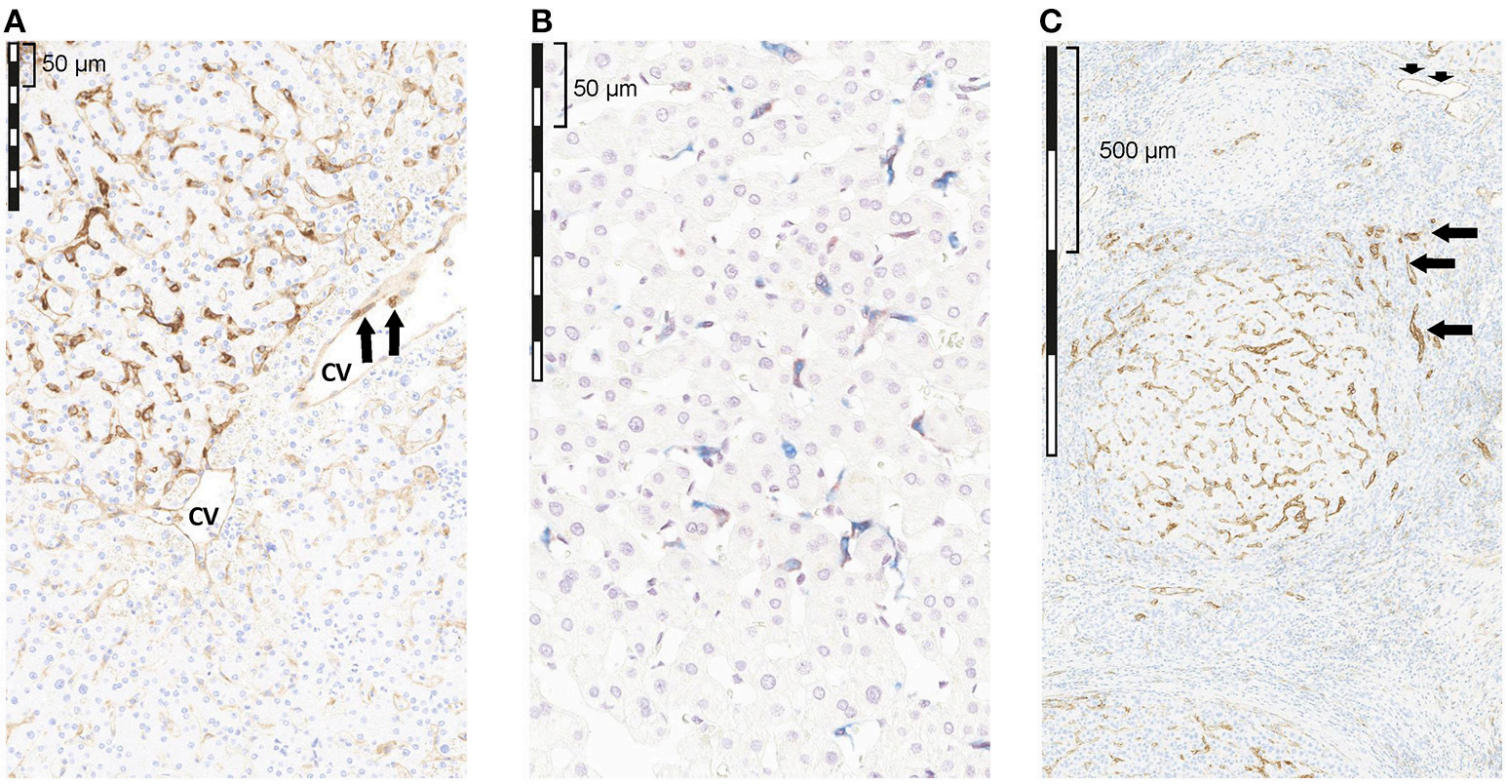
Qualitative assessment of PD-L1 expression by IHC in tumor-free liver adjacent to HCC with reactive changes. **(A)** non-cirrhotic liver, PD-L1 (brown); **(B)** non-cirrhotic liver, PD-L1 (brown) and CD68 (blue); **(C)** cirrhotic liver, PD-L1 (brown). **(A)** PD-L1 staining was seen in sinusoidal lining cells, as well as in ECs of a CV. The extent of PD-L1 staining varied considerably regionally as well as from case to case. **(B)** Double staining of PD-L1 (brown) and CD68 (blue) revealed that some, but not all, sinusoidal macrophages (Kupffer cells) in the TFL showed PD-L1 IR; however, the extent of PD-L1 staining in CD68^+^ cells varied considerably regionally as well as from case to case **(C)** in the cirrhotic liver tissue, membrane and/or cytoplasmic PD-L1 staining was present in the sinusoidal lining cells, ECs of microvessels (short arrows) and ECs of CVs, and in ICs. Hepatocytes were PD-1 negative. In contrast, the epithelium of bile ductules showed cytoplasmic and membranous PD-L1 staining (arrows). cv, central vein.

IHC analysis of PD-L1 expression showed that membrane and/or cytoplasmic PD-L1 staining was present in sinusoidal lining cells (8/8 cases, Figure 1A), endothelial cells (ECs) of microvessels and of central veins (6/8 cases, Figure 1A), and ICs in the liver tissue and in the blood vessels sometimes sticking to PD-L1^+^ ECs (8/8 cases, Figure 1A arrows). Hepatocytes were consistently PD-L1^−^ (8/8 cases). Double labeling of PD-L1 and CD68 showed that PD-L1^+^ cells along the sinus were mainly Kupffer cells (CD68^+^; Figure 1B). The extent of PD-L1 staining varied considerably between different regions of the same section, and from case to case. For example, in some regions of the same section (see Figure 1) and in different cases, Kupffer cells were strongly positive, whereas in others, Kupffer cells did not stain at all (Figure 1A). In the remaining eight cases with cirrhotic liver tissue, membrane and/or cytoplasmic PD-L1 staining was present in the sinusoidal lining cells of the cirrhotic tissue (Figure 1C), ECs of microvessels (Figure 1C short arrows) and ECs of central veins (6/8 cases), and in the ICs (8/8 cases). Hepatocytes were PD-L1^−^. By contrast, the epithelium of bile ductules showed cytoplasmic and membranous PD-L1 staining (Figure 1C arrows).

Unfortunately, in the remaining 52 cases extratumoral tissue was not available for evaluation.

**Figure 2:**
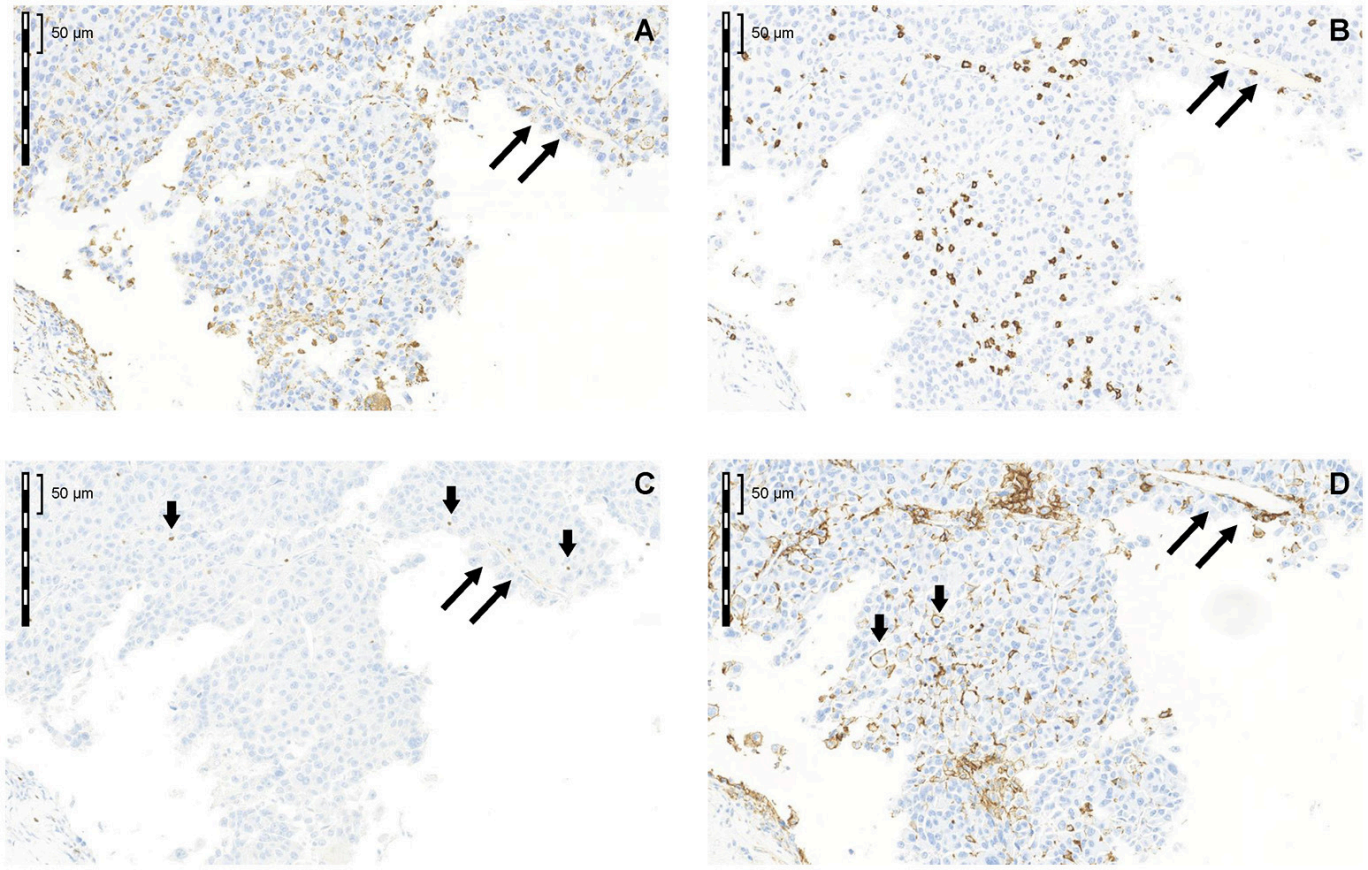
Cellular composition of a typical HCC in serial sections related to PD-L1 expression in this area **(A)** CD68; **(B)** CD8; **(C)** FoxP3; **(D)** PD-L1. Long arrows label a blood vessel that is present on every slide indicating that consecutive sections were stained. Of note, there are only a few scattered FoxP3^+^ cells, which were greatly outnumbered by CD8^+^ and CD68^+^ cells.

**Figure 3:**
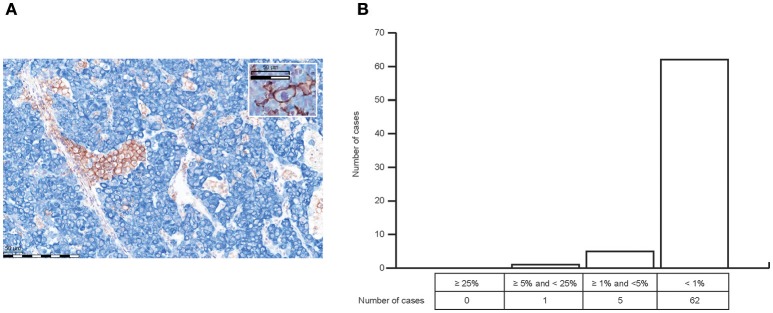
**(A)** PD-L1/cytokeratin double labeling (PD-L1 = brown; cytokeratin [clone AE1/AE3] = blue) confirmed that only a few tumor cells showed PD-L1 staining (inset **A**) Most PD L1^+^ cells in HCC were cytokeratin-negative, and were therefore ICs. **(B)** Semiquantitative analysis of PD-L1 expression in HCC revealed that only 6 of 68 HCC specimens (8.8%) were positive for PD-L1 staining in tumor cells with regard to the ≥1% cut-off.

Cytoplasmic and/or membrane PD-L1 staining in tumor cells and/or ICs was present within HCC in all 68 cases (Figure 2A). Levels of PD-L1 positivity did not correlate with histological subtype. PD-L1 monolabeling showed that it was sometimes difficult to safely discriminate between a PD-L1^+^ tumor cell and a PD-L1^+^-activated immune cell (Figure 2D short arrows). However, PD-L1/cytokeratin double labeling confirmed that only a few tumor cells showed PD-L1 staining (Figure 3). Indeed, semiquantitative assessment showed that only 6/68 HCCs (9%) were classified as PD-L1^+^ (≥1% of tumor cells). The incidence of ICs positive for CD8, CD68, and FoxP3 was highly variable between different cases (Figures 2B–D). ICs positive for each marker were more frequent in the invasive margin (whenever present) than in the center of the tumor. Figure 2 shows the cellular composition of a typical HCC on serial sections (long arrows indicate a blood vessel that is present on every slide indicating that consecutive sections were stained) related to PD-L1 expression in this area. Of note, there are only a few scattered FoxP3^+^ cells which were greatly outnumbered by CD8^+^ and CD68^+^ cells. Figure 3A shows that most PD-L1^+^ cells in HCC were cytokeratin-negative and were therefore ICs. Occasional PD-L1^+^ cytokeratin^+^ tumor cells were seen (Figure 3A, inset). As shown in Figure 3B, assessment of PD-L1 staining in tumor cells revealed that only 6 of 68 HCC specimens (8.8%) were positive for PD-L1 (≥1% cut-off). We were able to semi-quantitatively assess the tumor/stromal area occupied by PD-L1^+^ ICs in 43 specimens (due to technical reasons, not all 68 specimens were available for analysis). The area occupied by PD-L1-positive ICs was highly variable (median 2%), and in most (41/43) cases it was <25%, indicating that only a small number of HCCs have a high content of PD-L1^+^ ICs (data not shown).

**Figure 4:**
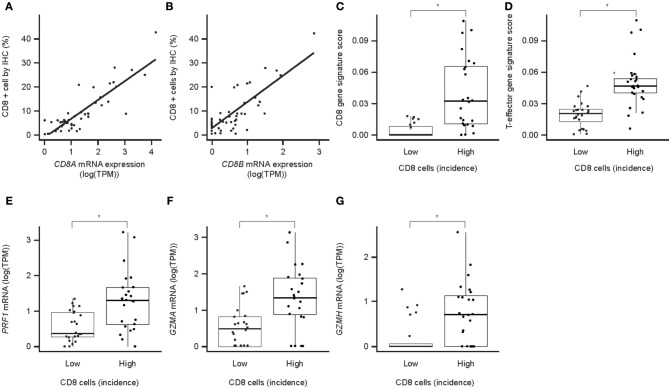
CD8 protein expression (IHC) and associated mRNA expression in HCC. **(A)** Correlation of CD8a mRNA expression with the number of CD8^+^ cells in IHC (*P* = 2.7 × 10^−16^); **(B)** correlation of CD8b mRNA expression with the number of CD8^+^ cells in IHC (*P* = 7.3 × 10^−11^); **(C)** association of CD8 T-cell GES with the number of CD8-positive cells in IHC; **(D)** association of T-effector-IFN-γ-associated GES with the number of CD8^+^ cells in IHC; **(E)** association of perforin mRNA with the number of CD8^+^ cells in IHC; **(F)** association of granzyme A mRNA with the number of CD8^+^ cells in IHC; **(G)** association of granzyme H mRNA with the number of CD8^+^ cells in IHC. Asterisk indicates *P* < 0.05. TPM, transcripts per million; PRF1, perforin 1; GZMA, granzyme A; GZMH, granzyme H.

Evaluation of IC infiltration by IHC revealed that, across samples, the incidence of CD8^+^ cells correlated with CD8A and CD8B mRNA levels (Figures 4A,B), and with the strength of a CD8 gene signature from CIBERSORT (Supplementary Figure 1). The incidence of CD8^+^ cells also correlated with the strength of a CD8 T cell GES (Figure 4C), suggesting CD8 T cell GES and/or CD8 mRNA expression by RNAseq may serve as an alternative approach to assessment of CD8 expression. Samples with a high incidence of CD8^+^ cells also had strong T-effector-IFN-γ-associated GESs (Figure 4D) and high levels of mRNA for the key cytotoxic factors perforin (PRF1), granzyme A (GZMA), and granzyme H (GZMH) (Figures 4E–G). Together, these findings suggest that PD-L1 is infrequently expressed in neoplastic hepatocytes and that PD-L1 staining in HCC is largely restricted to immune cells. CD8 staining and gene expression analysis may imply a pre-existing immunity observed in these samples.

### TGF-β in HCC Samples

**Figure 5:**
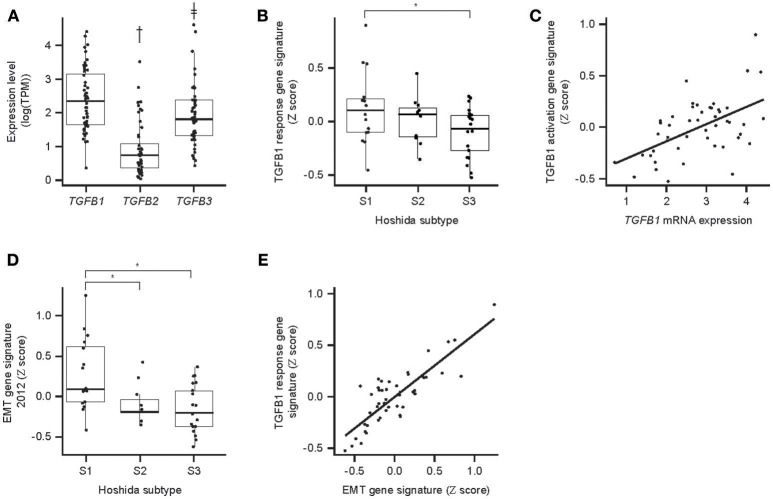
TGF-β expression and associated GES activity in HCC. **(A)** Expression of TGFB mRNA isoforms determined by RNAseq; **(B)** association between Hoshida molecular HCC subtype and TGF-β response-associated GES; **(C)** correlation between TGFB1 mRNA expression and the TGF-β1 response-associated GES (*P* = 7.9 × 10^−14^); **(D)** association between Hoshida molecular HCC subtype and EMT-associated GES; **(E)** correlation between TGF-β response-associated GES and EMT-associated GES (*P* = 7.9 × 10^−14^). An asterisk indicates *P* < 0.05; ^†^fold-difference 5.5, *P* = 6.4 × 10^−16^; ^‡^fold-difference 2.3, *P* = 0.008. EMT, epithelial/mesenchymal transition.

TGF-β gene expression and activity were evaluated in HCC samples. TGF-β activity via TGF-β mRNA levels of TGF-β genes in 48 HCC samples were determined from RNAseq data. All isoforms were detectable, with *TGFB1* being the most abundant (Figure 5A). Molecular profiling of 48 HCC samples using Hoshida's approach (26) showed that the S1 HCC subtype has a trend toward increased TGF-β1 activity-associated GES and increased EMT GES (Figure 5B). The TGF-β1 activity-associated GES strongly correlates with TGFB1 mRNA expression (Figure 5C). The strength of the EMT GES also differed by Hoshida subtype and was strongest in subtype S1 (Figure 5D). The TGF-β activity-associated GES and EMT GES were consistently strongly correlated (Pearson coefficient 0.84) (Figure 5E). Notably, these signatures comprise 229 genes and 59 genes, respectively, and the correlation remained when the seven genes in common were excluded from the signatures (Pearson coefficient with overlapping genes included: 0.84, *P* = 7.9^−14^; with overlapping genes removed: 0.63, *P* = 2.0^−06^).

### Relationships Between PD-L1, ICs, and TGF-β1

**Figure 6:**
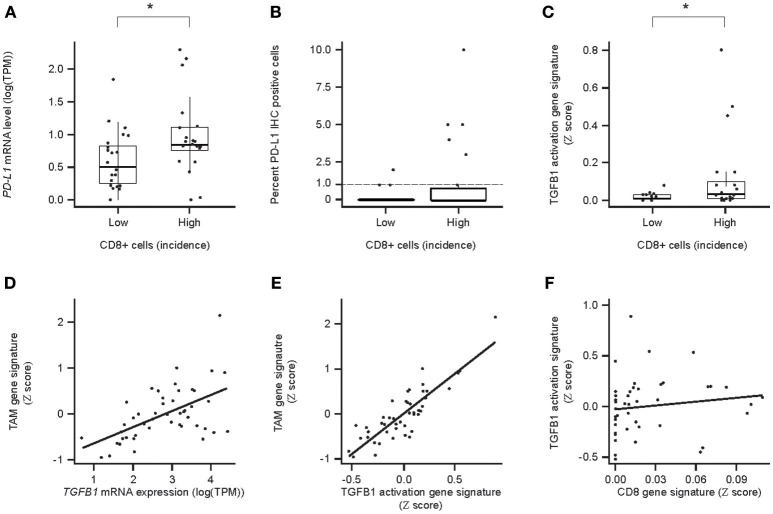
Relationships between PD-L1/PDL1/, CD8/CD8a expression, and TGF-β TGFB1/TGFb1 response/immune cell (IC) infiltrate-associated GES and TGF-β1 and tumor-associated ICs in HCC. **(A)** Association between PDL1 mRNA levels and the incidence of CD8^+^ cells. High and Low incidence was defined as above and below the median, respectively; **(B)** association between PD-L1^+^ and the incidence of PD-L1^+^ tumor cells and the incidence (Low or High) of CD8^+^ cells; **(C)** association of TGF-β response-associated GES between PD-L1^+^ ICs and the incidence (Low or High) of CD8^+^ cells; **(D)** correlation between TGFB1 mRNA levels and tumor associated macrophage (TAM) -associated GES; **(E)** correlation for the strength of GESs associated with the TGF-β1 activity response and TAMs (*P* = 7.0 × 10^−14^); **(F)** correlation between a CD8-associated GES and a TGF-β1-activation response-associated GES (*P* = 5.9 × 10^−1^). CD8 IHC High and Low defined as above and below the median, respectively. An asterisk indicates *P* < 0.05. TAM, tumor-associated macrophages.

Correlation analyses were performed to determine the relationship between PD-L1, ICs, and TGF-β1. PD-L1 (*CD274*) mRNA levels correlated with the incidence of CD8^+^ cells across the HCC samples (Figure 6A). Despite low levels of PD-L1 IHC, a trend toward correlating with CD8 IHC was seen, especially for PD-L1 staining in ICs (Figures 6B,C).

*TGFB1* mRNA levels and the strength of the TGF-β activation-associated GES also correlated with the strength of the TAM GES (Figures 6D,E). As a control, we showed that there was no correlation between TGF-β1-associated and CD8 T cell-associated GESs, which are expected to be unrelated (31) (Figure 6F).

## Discussion

The primary objectives of this observational study were to investigate the expression of PD-L1 in HCC and adjacent non-tumor liver, the frequency and identity of ICs in HCC, and the expression/activity of TGF-β1 in HCC. The purpose was to gain a greater understanding of the immunogenic environment associated with HCC, including the TME and infiltrating ICs, building on previously published research (12, 13). To achieve these objectives, histologic, IHC, and RNAseq data, generated for a set of HCC samples, were analyzed descriptively. Our results showed that CD8 gene expression correlates with the number of CD8^+^ cells (assessed by quantitative IHC) in HCC tissue samples. Importantly, TGF-β expression and activation shows a strong correlation with the strength of TAM activity GES. In addition, PD-L1 expression is largely restricted to IC. Thus, PD-L1^+^ ICs and TGF-β activity are features of HCC that likely play a role in immune evasion.

PD-L1 is implicated in immune suppression in HCC by its presence in tumors and adjacent tissue, and high PD-L1 expression in HCC has been positively correlated with liver cirrhosis, poor Barcelona Clinical Liver Cancer stage, portal vein invasion, and reduced overall survival (32). We found that PD-L1 is detectable by IHC in non-tumor liver tissue, and also in cirrhotic liver tissue. It is mainly located in immune cells (e.g., Kupffer cells), and also in endothelial cells of central veins and microvessels in the non-cirrhotic and cirrhotic liver tissue, as well as in intratumoral microvessels. In HCC, PD-L1 staining is rare on tumor cells and occurs mostly on infiltrating ICs. These findings differ from those of Calderaro et al., who, while identifying PD-L1 most frequently on immune cells, also found that 17% of HCC tumor cells expressed PD-L1 (13), and Yarchoan et al., who identified PD-L1-positive cell clusters in 24/29 tumors (11). However, as these studies did not appear to differentiate tumor and immune cells, it is possible that the incidence of PD-L1^+^ tumor cells was overestimated. CD8^+^, CD68^+^, and FoxP3^+^ cells were found to occur with variable incidences in HCC, with the highest numbers at the invasive margin, suggesting greatest immunological activity in this region. However, as shown by semiquantitative IHC, the content of PD-L1^+^ immune cells in HCC is generally low. The expression of PD-L1 in HCC primarily on infiltrating ICs rather than tumor cells suggests that tumors may evade immune surveillance primarily by manipulation of PD-L1^+^ ICs, e.g., cytotoxic T lymphocytes. Additionally, PD-L1 expression by endothelial cells in small blood vessels, central veins, and intratumoral microvessels may have a role in immune regulation. Liver sinusoidal endothelial cells (and hepatocytes) have previously been shown to act as unconventional antigen presenting cells (APCs) in the liver, so when expressing PD-L1 they may anergize infiltrating ICs (33). Speculatively, PD-L1^+^ endothelial cells may also block the transmigration of PD-1^+^ ICs into the liver (34).

We found that the incidence of CD8^+^ cells correlated with higher levels of mRNAs for perforin, granzyme A, and granzyme H. We also found a correlation between the incidence of PD-L1^+^ cells and the incidence of CD8^+^ cells and the strength of a CD8-associated GES, suggesting that PD-L1 expression may increase as an adaptive anti-inflammatory response to pre-existing CTL activity (25, 35).

IHC is an established method for detecting clinically relevant expression of PD-L1 in HCC (13, 36) and expression of CD8, CD68, and FoxP3 on ICs (37). Measures of TGF-β1-mediated immune suppression, however, are not well-established. We did not attempt to assess TGF-β1 expression in HCC by IHC, because soluble protein is liable to be lost during tissue fixation and processing, and appropriate validated antibodies are unavailable. Instead, we confirmed the presence of *TGFB1, TGFB2*, and *TGFB3* transcripts in HCCs using RNAseq data. However, we considered that expression levels of individual isoforms of *TGFB* mRNA may be poor indicators of TGF-β activity, because activity is also dependent on availability of mature and free TGF-β ligands, turnover, and cellular context. We therefore additionally used a previously described TGF-β1-associated GES as a measure of TGF-β activity in HCC. Our results demonstrated that TGF-β1 mRNA is detectable in HCC.

The observed correlation between TGF-β1- and EMT-associated GESs is consistent with the established role of TGF-β in the induction of EMT in HCC (38), and indicates that levels of TGF-β mRNA detectable in HCC have significant biological activity. Levels of TGF-β1 were higher than levels of TGF-β2 and TGF-β3, suggesting that it may be the most important isoform affecting HCC. The TGF-β1-associated GES correlated with a TAM-associated GES, which is consistent with TAMs being a significant source of TGF-β1 in the liver (39). It is important to note, however, that TGF-β1 may also be produced by liver endothelial cells and Kupffer cells (40). The potential contribution of these sources to TGF-β1 activity in this series of HCCs is unknown.

Only one section per patient was available for analysis in this study, as evidenced by the relatively low proportion of patients with adjacent non-neoplastic liver parenchyma. Since heterogeneity in this setting is an issue, this is acknowledged as a limitation to this study. In addition, as this study was executed on procured tissue samples, and clinical characteristics were not available, it was not possible to correlate tissue histology with clinical characteristics, such as cirrhosis and clinical behavior.

Our study contributes to the hypothesis that inhibition of PD-L1 and TGF-β and their respective immunomodulatory pathways may contribute to antitumor effects in HCC. Our observations are consistent with those of others who have identified roles for PD-L1, TGF-β1, and infiltrating ICs in HCC (1, 5, 12, 13, 36, 41). Our work extends previous research by assessing these parameters in a series of HCCs, albeit based on observational studies, and by combining IHC and RNAseq data analysis to gain insight into complementary mechanisms of immunosuppression in HCC. Validation of these findings is needed in an additional cohort of patients lacking HCC.

Our results suggest that inhibiting both PD-L1 and TGF-β may increase antitumor immunity beyond that achievable by inhibiting either in isolation. Therefore, there may be an opportunity to combine PD-1/PD-L1 inhibitors with TGF-β-inhibiting compounds to improve the activity of immuno-oncology treatment approaches.

## Data Availability Statement

Merck KGaA operates a responsible Data Sharing Policy and requests for access to the datasets for this study can be made directly to Merck in line with this policy.

## Author Contributions

CI, YZ, ID, LT, and BN contributed to the concept and design of the study. PR and EF-K contributed to experiments and procedures. All authors contributed to the writing of the manuscript.

### Conflict of Interest Statement

CI and EF-K are employees of Merck KGaA. BN, YZ, PR, and ID are employees of EMD Serono. LT is a consultant pathologist for Merck KGaA.
