# Supplementary material for: Observational Study of PD-L1, TGF-β, and Immune Cell Infiltrates in Hepatocellular Carcinoma
Source: Front Med (Lausanne). 2019 Feb 8;6:15. doi: 10.3389/fmed.2019.00015 (PMC6375852; doi:10.3389/fmed.2019.00015)
Supplement: Supplementary file 1 [file Data_Sheet_1.DOCX]

**Supplementary Table S1**. Patient and tumor characteristics of liver samples included in this study.

| Patient ID | Age | Sex | Histological diagnosis  Prevalent growth pattern | Grading  (Edmondson-Steiner) |
| --- | --- | --- | --- | --- |
| 1 | 42 | Female | HCC solid_trabecular | 2 |
| 2 | 31 | Female | HCC solid_trabecular | 3 |
| 3 | 66 | Female | HCC clear-cell type | 2 |
| 4 | 37 | Male | HCC solid_trabecular | 3 |
| 5 | 64 | Male | HCC solid_trabecular | 4 |
| 6 | 83 | Female | HCC clear-cell type | 3 |
| 7 | 45 | Male | HCC solid_trabecular | 3 |
| 8 | 59 | Female | HCC solid_trabecular | 3 |
| 9 | 31 | Male | HCC solid_trabecular | 4 |
| 10 | 51 | Male | HCC solid_­pseudoglandular | 3 |
| 11 | 29 | Male | HCC solid | 2 |
| 12 | 49 | Male | HCC solid_trabecular | 3 |
| 13 | NA | NA | HCC solid | 2 |
| 14 | 59 | Male | HCC solid | 4 |
| 15 | NA | NA | HCC solid_­pseudoglandular | 2 |
| 16 | 51 | Male | HCC solid | 4 |
| 17 | 32 | Male | HCC solid­_pseudoglandular | 2 |
| 18 | 45 | Male | HCC solid | 4 |
| 19 | 50 | Male | HCC solid | 3 |
| 20 | NA | NA | HCC solid | 3 |
| 21 | NA | Male | HCC solid_trabecular | 3 |
| 22 | 72 | Male | HCC solid_­pseudoglandular | 3 |
| 23 | 47 | Male | HCC solid_trabecular | 3 |
| 24 | 35 | Male | HCC solid_trabecular | 3 |
| 25 | 36 | Female | HCC solid_trabecular | 3 |
| 26 | 46 | Male | HCC solid_trabecular | 2 |
| 27 | 70 | Male | HCC solid_trabecular | 3 |
| 28 | 38 | Male | HCC solid_trabecular | 3 |
| 29 | 49 | Male | HCC solid_trabecular | 3 |
| 30 | 17 | Female | HCC solid_trabecular | 4 |
| 31 | 61 | Male | HCC solid_trabecular | 3 |
| 32 | NA | NA | HCC solid_trabecular | 3 |
| 33 | 58 | Male | HCC solid_trabecular | 2 |
| 34 | 40 | Male | HCC solid_trabecular | 3 |
| 35 | 64 | Male | HCC solid_trabecular | 2 |
| 36 | 77 | Male | HCC solid_pseudoglandular | 2 |
| 37 | 60 | Male | HCC solid_trabecular | 4 |
| 38 | 68 | Female | HCC solid_trabecular | 3 |
| 39 | 45 | Male | HCC solid_trabecular | 2 |
| 40 | 47 | Male | HCC solid_­pseudoglandular | 2 |
| 41 | 45 | Male | HCC solid_trabecular | 3 |
| 42 | 51 | Male | HCC solid_trabecular | 2 |
| 43 | 70 | Male | HCC solid_trabecular | 2 |
| 44 | 76 | Female | HCC solid_trabecular | 2 |
| 45 | 57 | Male | HCC solid_trabecular | 3 |
| 46 | 70 | Female | HCC solid_trabecular | 4 |
| 47 | 30 | Male | HCC solid_trabecular | 3 |
| 48 | 76 | Male | HCC solid_trabecular | 3 |
| 49 | 67 | Male | HCC, solid­_pseudoglandular | 2/3 |
| 50 | 78 | Male | HCC solid_trabecular | 3 |
| 51 | 63 | Male | HCC solid_trabecular | 3 |
| 52 | 50 | Male | HCC solid_pseudoglandular | 2 |
| 53 | 67 | Male | HCC solid_trabecular | 3 |
| 54 | 56 | Male | HCC solid_trabecular | 2/3 |
| 55 | 60 | Male | HCC solid_trabecular | 4 |
| 56 | 29 | Female | HCC solid_trabecular | 3 |
| 57 | 67 | Female | HCC solid_trabecular | 3 |
| 58 | 75 | Male | HCC solid_trabecular | 3 |
| 59 | 60 | Male | HCC solid_pseudoglandular | 2 |
| 60 | 71 | Male | HCC solid_trabecular | 3 |
| 61 | 64 | Male | HCC desmoplastic | 3 |
| 62 | 73 | Male | HCC solid_trabecular | 2 |
| 63 | 91 | Male | HCC solid_trabecular | 3 |
| 64 | 66 | Male | HCC solid_trabecular | 2 |
| 65 | 59 | Female | HCC solid_trabecular | 4 |
| 66 | 65 | Male | HCC solid_trabecular | 3 |
| 67 | 66 | Male | HCC solid_trabecular | 3 |
| 68 | 71 | Male | HCC solid_trabecular | 3 |

**Supplementary Table S2.** Genes comprising gene signatures.

| Signature | Genes |
| --- | --- |
| **Huang2012_EMTup** | ADAM23, ADAMTS1, AFF3, AHNAK, AK5, AKAP12, ALPK2, ANGPTL2, ANKRD1, ANTXR1, ANXA6, AOX1, AP1S2, ARMCX1, ATP8B2, ATP8B3, AXL, BDNF, BGN, BICC1, BIN1, BMP1, BNC2, BVES, C16orf45, C1S, C5orf13, C9orf30-TMEFF1, CALD1, CAMK2N1, CAP2, CCDC92, CCL2, CDH11, CDH2, CDH4, CDKN2C, CHN1, CLDN11, CLIP3, CMTM3, COL12A1, COL1A2, COL3A1, COL4A1, COL5A1, COL5A2, COL6A1, COPZ2, CREB3L1, CTGF, CYBRD1, DAB2, DCN, DDR2, DFNA5, DIO2, DKK3, DLC1, DLEU7, DNAJB4, DOCK10, DPT, DPYSL3, ECM1, EDIL3, ELOVL2, EMP3, ENOX1, ENPP2, EPB41L5, EPDR1, EVI2A, F2R, FADS2, FAM101B, FAP, FAT4, FBLN1, FBLN5, FBN1, FGF2, FGF5, FGFR1, FHL1, FILIP1L, FLRT2, FN1, FOXC2, FSTL1, GFPT2, GLIPR1, GLT25D2, GNB4, GNG11, GPC6, GPR176, GREM1, GSC, HAS2, HEG1, HS3ST2, HS3ST3A1, HTRA1, IGFBP3, IGFBP4, IGFBP7, IL13RA2, ITGA5, ITGAV, JAM3, KIRREL, KRT81, LAMA4, LGALS1, LHFP, LIX1L, LMCD1, LOX, LTBP1, LTBP2, MAP1B, MLPH, MME, MMP1, MMP2, MMP3, MMP9, MRAS, MSN, MSRB3, MYL9, NAP1L3, NAV3, NDN, NEBL, NEGR1, NEXN, NID1, NR2F1, NRG1, NRP1, NUDT11, OLFML3, PAPPA, PCOLCE, PDE7B, PDGFC, PLAGL1, PMP22, PNMA2, POPDC3, POSTN, PPAP2B, PPM1D, PRKD1, PRR16, PRRX1, PTGER2, PTGIS, PTRF, PTX3, PVRL3, RBM24, RBMS3, RBPMS2, RECK, RFTN1, RGL1, RGS4, ROR1, SCCPDH, SEMA5A, SEPT6, SERPINE1, SIRPA, SLC2A3, SLC47A1, SNAI1, SNAI2, SNAI3, SOX10, SPARC, SPOCK1, SRGN, SRPX, ST3GAL2, STARD13, STC1, STEAP1, SUSD5, SYDE1, TAGLN, TBX3, TBXA2R, TCF4, TGFB1I1, TGFB2, THY1, TIMP1, TMEFF1, TMEM132A, TMEM158, TMEM47, TNFAIP6, TNFRSF19, TNS3, TPM1, TRAM2, TRPA1, TSHZ1, TTC28, TTLL7, TUB, TUBA1A, TUBA1B, TWIST1, UGDH, VCAN, VIM, VPS13A, WIPF1, WNT5A, WNT5B, XYLT1, ZBTB38, ZEB1, ZEB2, ZFPM2 |
| **genentech.teff** | CD8A, CXCL10, CXCL9, EOMES, GZMA, GZMB, IFNG, PRF1, TBX21 |
| **ipa.tgfb1.activated** | ABL1, ACTA2, ADAM17, AKT1, ATF2, CASP3, CASP9, CDH1, CDK4, CFL1, COL1A2, COL3A1, COL6A1, COL6A3, CTCF, CTGF, CTNNB1, E2F1, EGFR, ENG, ERBB2, FAS, IGF2R, JUN, MAP2K3, MAP3K7, MAPK1, MAPK13, MAPK14, MAPK3, MAPK8, MMP1, MMP10, NFKB, NOTCH1, NR2C2, NUAK1, PAI, PAK2, PLAU, PRKAA1, PTGS2, RHOA, RHOB, RPS6KA1, S100A4, SERPINE1, SMAD1, SMAD2, SMAD3, SMAD5, SMAD7, SPHK1, SRC, STAT6, TGM2, TIMP1, TP53, ZFP36 |
| **Finkernagel2016.TAM.up** | ACE, ADSSL1, APOE, ARMC9, CA12, CCL18, COL1A1, COL1A2, COL3A1, COL5A1, COL5A2, CTD-2501B8.1, EDNRB, FAM177B, FHAD1, FLNC, LOX, LUM, MMP19, PAPPA, PCOLCE2, PDGFRA, PIEZO2, PTGIS, SERPINE1, SLC16A10, SPP1TMEM26, TPST1, TSPAN13 |
| **hoshida2009.HCC.S1** | IQGAP1, S100A11, RAB31, CD37, POSTN, ARHGDIB, ALOX5AP, LAPTM5, CSPG2, ARPC2, IFI30, COL1A2, GPNMB, TMSB4X, ANXA1, LUM, CDK2AP1, LHFPL2, LSP1, CUGBP2, CTSC, LCP1, IER3, PTPRC, COL3A1, MSN, SLA, IL2RG, COL4A2, GEM, PLP2, ARPC1B, CYP1B1, MGP, ANXA5, DAB2, TAX1BP3, PRG1, C3AR1, CCL5, PYGB, TPM2, COL4A1, CBFB, THY1, ALDOA, ITPR3, BLVRA, HK1, CD53, HLA-DQB1, CORO1A, PLAUR, TIMP2, OAZ1, HLA-DPB1, IL7R, BCL2A1, TRAF3, LAMB1, NCF1, LTBP2, CDH11, SLC1A5, KLF5, LDHB, COL15A1, TCF4, CD47, CTGF, NP, ID3, IGLL1, KIAA0101, FBN1, ADAM8, HIF1A, TRAF5, COL6A1, ACTA2, LGALS1, TSPAN3, PFN1, NSMAF, ATP6V1F, LTBP3, CYBB, HLA-DQA2, LGALS3BP, NPC2, EFEMP1, MAP1B, TAGLN, ATP6V1B2, DCTN2, CXCR4, HLA-DMA, FLNA, FCGBP, MYCBP2, PPIC, AEBP1, HCLS1, PAM, S100A10, F13A1, C1QB, GUCY1A3, LMO4, STX3, STK38, EFNB1, CAPZA1, MPHOSPH6, ZNF384, CD74, NBL1, CD151, MTHFD2, GRN, RNASE6, CRABP2, CDC25B, DDR1, PKN1, IFI16, SLC39A6, PKMYT1, IKBKE, COL6A2, GYPC, CYR61, ADAM9, ASAH1, GSTP1, CRIP2, TRA@, COL5A2, PIM2, COL11A1, IGH@, DPYSL2, QSCN6, CSRP3, FCGR2A, GNAI2, RALGDS, ACP5, FYB, IGKC, LITAF, POLD3, RSU1, PEA15, FHL3, ANXA4, CD3D, M6PR, RNASE1, KNS2, IGFBP5, PKD2, CTSS, FBS1, DGKA, SLC2A5, SLC7A5, FUT4, CDC20, CYBA, PRKD2, TP53BP1, S100A13, DUT, IRF1, SMARCD1, AQP1, PAK1, CCR7, TRIP10, LYN, ARF5, FGL2, CXCL1, GLIPR1, PSMD2, CCND2, AIF1, PAPSS1, SRI, ME1, PLD3, DUSP5, DNM2, ADAM15, HLA-DOA, GNS, RCC1, RIT1, PPP4R1, RHOA, MFAP1, ATP1B3, PROCR, ATP6AP1, DDX11, LOX, LGMN, CD48, ITGB2, TFF3, DGKZ, RIN2, SLBP, CD8A, RPA2, SPAG8, CYFIP1, TNFRSF1B, RALB, IL2RB, PGK1, CCL3, PPP1CB, IL15RA, SLC2A1, AP2S1, LTF, TUBA1, AKT3, PRMT5, LGALS9, ATP6V0B, HEXA, SMAD2, CHN1, DDR2, PPP4C |

**Supplementary Fig. S1.** Correlation between CD8 expression by IHC and CD8-associated activity by GES.
